# Vitamin D3 regulates apoptosis and proliferation in the testis of D-galactose-induced aged rat model

**DOI:** 10.1038/s41598-019-50679-y

**Published:** 2019-10-01

**Authors:** Malsawmhriatzuala Jeremy, Guruswami Gurusubramanian, Vikas Kumar Roy

**Affiliations:** Department of Zoology, Mizoram University, Aizawl, Mizoram 796 004 India

**Keywords:** Ageing, Reproductive biology

## Abstract

The age-associated imbalances between proliferation and apoptosis lead to impaired spermatogenesis and infertility. The age-associated decline in vitamin D3 levels has been reported and suggested the anti-aging potential of vitamin D3. However, the age-associated decline levels of vitamin D3 has not been studied in relation to the testicular activity. Thus, we investigated the effect of vitamin D3 on the expression of testicular proliferation markers, apoptotic markers, antioxidants system and oxidative stress in a D-gal-induced aged rat model. The present study investigated the levels of vitamin D3 and AGE in serum and testes along with the expression of the AGE-receptor (AGER) in the testis. Vitamin D3 treatment significantly increases cell proliferation and decreases apoptosis in a D-gal-induced aged rat testis. Furthermore, vitamin D3 significantly decreases oxidative stress in aged rat testis by improving the antioxidant defense systems. The expression of AGER was down-regulated by vitamin D3 treatment in aged testis. The circulating and intra-testicular AGE was higher in aged groups, however, only circulating vitamin D3 levels decreased in aged groups. The immunolocalization of VDR showed increased immunostaining in the testis by vitamin D3 treatment. Thus, it can be concluded that vitamin D3 delays testicular senescence by regulating proliferation and apoptosis.

## Introduction

Vitamin D3 is traditionally known for its action for maintaining and regulating calcium and phosphorus homeostasis. Apart from its general impact on mineral metabolism, vitamin D3 acts as an important endocrine hormone, which plays a significant role in modulating both male and female reproductive processes^1,2^. In the last decade, the relationship between vitamin D and reproduction has been increasingly recognized due to the presence of receptors for vitamin D (VDR) in both male and female reproductive organs^1^. It has also been shown that elderly persons are at higher risk of developing hypovitaminosis D due to the reduced vitamin D production potential in the skin with advancing age^3^. Recently, it has also been revealed that vitamin D deficiency is associated with erectile dysfunction, reduced sperm concentration and, reduced motility of sperm^1,4,5^. Despite human studies, various rodent studies have also suggested that reduced sperm counts, motility and reduced mating ability are associated with vitamin D deficiency^5^. The relationship between serum vitamin D3 and male fertility is not clear, however, there are reports suggesting vitamin D deficiency is one of the reasons for male infertility^6–8^. These findings were further supported by another study where lower fertility rate has been demonstrated in female rats inseminated from vitamin D deficient male rats^9^

Aging is a time-dependant gradual progressive deterioration, accompanied by deceleration of various physiological functions leading to increased vulnerability and mortality^10,11^. The exact underlying mechanism of aging has not been revealed. However, it is evident that the production of free radicals and lifespan are strongly correlated. It has been suggested that one of the mechanisms of aging could be the accumulation of reactive oxygen species (ROS) in aged person^12^. The reproductive potential also decreases with age, and this decline in reproductive capacity with age may result from a combination of morphological and molecular alterations in the reproductive organs^13^. It has been shown that advanced paternal age is associated with lower pregnancy due to poor sperm quality^14,15^. Furthermore, the decline of circulating vitamin D3 has also been reported in several aging-related pathophysiological conditions^16^. This decline of vitamin D has become a major health concern^17^ and suggested that the number of people suffering from vitamin D deficiency would increase and elderly people would be prevalent^18^. However, the relationship between decrease vitamin D3 levels and decrease reproductive capacity has not been investigated in the male subject. The role of vitamin D3 in testicular aging is yet to be investigated. It has also not been investigated whether the vitamin D3 treatment in the aged subject would affect the testicular activity or not.

A recent study revealed that some of the testis-specific genes can be up-regulated by vitamin D supplementation in mice^9^. The proliferation of skeletal muscle cells, endothelial cell proliferation and inhibition of apoptosis have been reported to be regulated by vitamin D^19,20^. Another study also suggests that the functional expression of antioxidant system alters in aging in male rat^21^. Furthermore, antioxidants (L-ascorbic acid, Zinc, and Selenium) have been shown to enhance germ cell survival and proliferation^22^. Advanced glycation end products (AGE) and their receptor (AGER) regulate various cellular processes and lead to increased oxidative stress and apoptosis^23^. Since vitamin D3 is also a well-known antioxidant, its role in testicular aging has not been explored in relation to germ cell proliferation and apoptosis. A recent study revealed that vitamin D deficiency impairs testicular development and spermatogenesis by inhibiting testicular germ cell proliferation^24^. Thus, it is logical to hypothesize that vitamin D3 might ameliorate age-associated testicular dysfunction in D-galactose treated animal model by regulating proliferation, apoptosis and antioxidant system. To the best of our knowledge, the present study will be the first to demonstrate the role of vitamin D3 in testicular proliferation and apoptosis in aged rodent animal model.

## Results

### Effect of vitamin D3 on expression and localization of GCNA and PCNA

**Figure 1:**
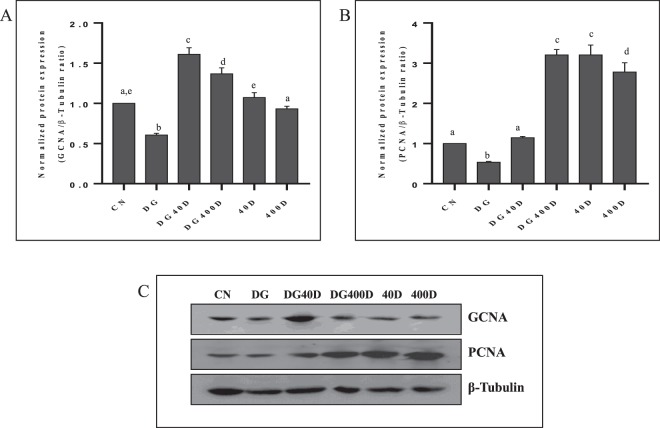
Vitamin D3 increased germ cell proliferation in D-gal-induced aged rat testis. (**A**) Densitometry of Western blots of GCNA protein expression in rat testis. (**B**) Densitometry of Western blots of PCNA protein expression in rat testis. (**C**) Representative Western blots of GCNA and PCNA protein expression in rat testis. Values are mean ± SEM (n = 6) and different superscripts show significant (*P* < 0.05) difference in protein expression. CN Control, DG D-galactose, DG40D D-galactose plus 40 IU/Kg vitamin D3, DG400D D-galactose plus 400 IU/Kg vitamin D3, 40D 40 IU/Kg vitamin D3, 400D 400 IU/Kg vitamin D3 groups. Cropped blots are shown. Uncropped blots are presented in Supplementary Fig. S1.

To clarify whether vitamin D3 would be involved in the regulation of testicular proliferation during aging, we have investigated the effect of vitamin D3 on GCNA and PCNA, which are well-known markers for cell proliferation. Western blot analysis showed a significant (*P* < 0.05) down-regulation of GCNA and PCNA in D-gal-induced aged rat testis (DG) compared to all other groups (Fig. 1A,B). Both dose of vitamin D3 (DG40D and DG400D) significantly (*P* < 0.05) up-regulated the expressions of GCNA and PCNA compared to the aged group (DG). Expression of GCNA showed highest in vitamin D3 treated aged rat testis (DG40D and DG400D), however alone treatment of vitamin D3 at either dose to normal rat (40D and 400D) was not statistically different from the control group (CN). PCNA showed high expression in aged rat testis treated with a high dose of vitamin D (DG400D and a normal rat treated with either dose of vitamin D3 (40D and 400D) compared to all other groups.

**Figure 2:**
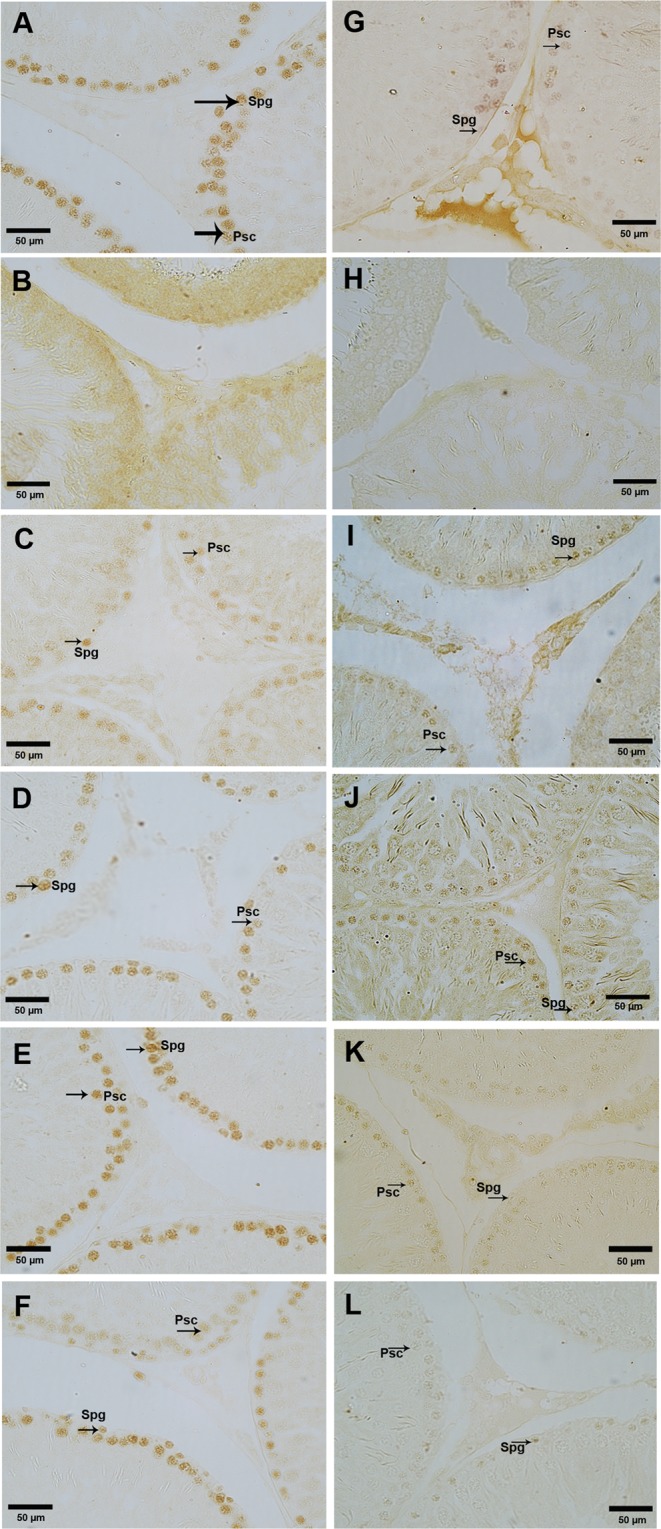
Effect of vitamin D3 on the localization of GCNA and PCNA in testis. A-F Localization of PCNA also showed its expression in spermatogonia (Spg) and spermatocytes (Psc) arrow) in the testis of control (**A**), D-gal-induced aged rat testis treated with vitamin D3 at dose of 40 IU/Kg (**C**), 400 IU/Kg (**D**), normal rat treated with alone vitamin D3 at dose of 40 IU/Kg (**E**) and 400 IU/Kg. (**F**) D-gal-induced aged rat testis showed no immunostaining of PCNA. (**B**) G-L Localization of GCNA confined to spermatogonia (Spg) and spermatocytes (Psc) (arrow) in the testis of control (**G**), D-gal-induced aged rat testis treated with vitamin D3 at dose of 40 IU/Kg (**I**), 400 IU/Kg (**J**), normal rat treated with alone vitamin D3 at dose of 40 IU/Kg (**K**) and 400 IU/Kg. (**L**) D-gal-induced aged rat testis showed no immunostaining of GCNA. (**H**) The figure shows a magnification of 40x.

Our immunohistochemical study also exhibits marked variation in the immunostaining of PCNA and GCNA. Localization of PCNA and GCNA were confined to spermatogonia and spermatocytes in control rat testis (Fig. 2A,G). In D-gal-induced aged rat testis, PCNA and GCNA showed no staining in spermatogonia and spermatocytes (Fig. 2B,H). The treatment of vitamin D3 to D-gal-induced aged rat resumption of PCNA and GCNA positive seminiferous tubules in the testis (Fig. 2C,D and Fig. 2I,J). Furthermore, vitamin D3 treatment to normal rat showed intense staining of PCNA in testis (Fig. 2E,F); however, in these groups, GCNA showed mild staining in the testis (Fig. 2K,L).

### Effect of vitamin D3 on the expression of anti-apoptotic (BCL2), apoptotic (BAX and active caspase-3) markers and TUNEL positive cells

**Figure 3:**
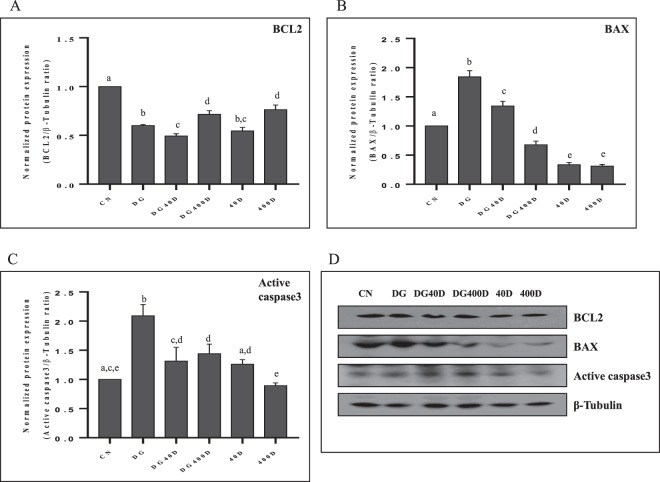
Vitamin D3 dependent expression of anti-apoptotic (BCL2) and apoptotic (BAX and active caspase-3) in the testis. (**A**) Densitometry of Western blots of BCL2 protein expression in rat testis. (**B**) Densitometry of Western blots of BAX protein expression in rat testis. (**C**) Densitometry of Western blots of active caspase-3 protein expression in rat testis. (**D**) Representative Western blots of BCL2, BAX and active caspase-3 protein expression in rat testis. Values are mean ± SEM (n = 6) and different superscripts show significant (*P* < 0.05) difference in protein expression. CN Control, DG D-galactose, DG40D D-galactose plus 40 IU/Kg vitamin D3, DG400D D-galactose plus 400 IU/Kg vitamin D3, 40D 40 IU/Kg vitamin D3, 400D 400 IU/Kg vitamin D3 groups. Cropped blots are shown. Uncropped blots are presented in Supplementary Fig. S1.

Since germ cell proliferation and apoptosis are processes, which take place simultaneous in the testis. Thus, we also investigated the expression of important markers for the regulation of apoptosis. BCL2 expression was significantly (*P* < 0.05) down-regulated in D-gal-induced aged rat testis (DG) and aged rats treated with either dose of vitamin D3 alone (DG40D and DG400D) compared to the control group (CN). However, the treatment of vitamin D3 at a dose of 40 IU/Kg to aged rat showed a significant (*P* < 0.05) decrease in BCL2 expression compared to aged rat (DG) and vitamin D3 at a dose of 400 IU/Kg significantly (*P* < 0.05) increased BCL2 expression compared to DG and DG40D. The alone treatment of vitamin D3 to aged rat showed a significant (*P* < 0.05) decrease in BCL2 expression compared to the control group (CN) (Fig. 3A).

The expression of BAX protein was significantly (*P* < 0.05) highest in D-gal-induced aged rat testis (DG) compared to other groups. The expression of BAX protein was significantly (*P* < 0.05) down-regulated by vitamin D3 in aged rat testis (DG40D and DG400D) compared to aged rat testis (DG). Vitamin D3 treatment to normal showed significantly (*P* < 0.05) decreased expression of BAX protein in the testis (40D and 400D) compared to other groups (Fig. 3B).

The active caspase-3, which is an executor of apoptosis, showed a significantly (*P* < 0.05) maximum expression in D-gal-induced aged rat testis (DG) compared to other groups. The expression of active caspase-3 showed significant down-regulation by vitamin D3 treatment to D-gal-induced aged rat testis at both doses (DG40D and DG400D) compared to aged group (DG). Vitamin D3 treatment to normal rat at dose of 40 IU/Kg (40D) showed significant (*P* < 0.05) up-regulation and at dose of 400 IU/Kg (400D) showed significant (*P* < 0.05) down-regulation of active caspase-3 in the testis compared to the control group (CN) (Fig. 3C).

**Figure 4:**
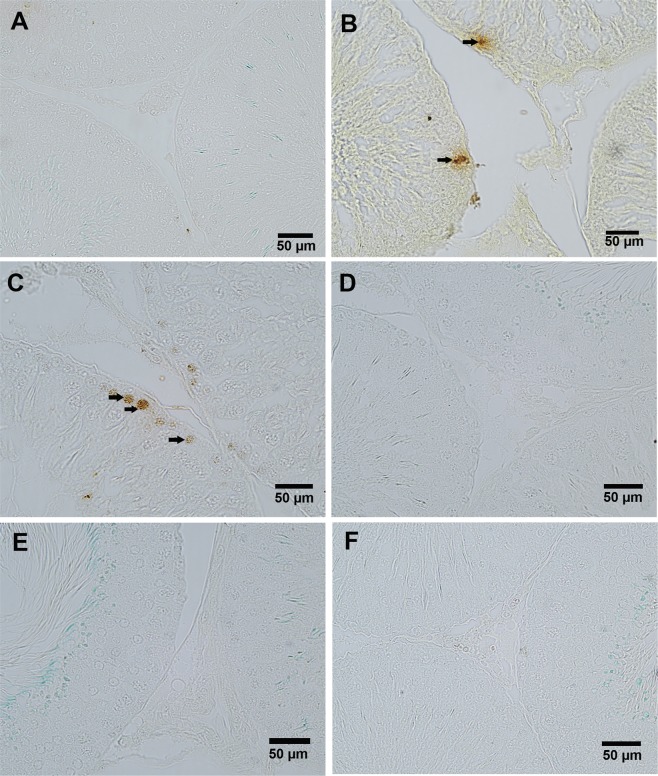
TUNEL immunohistochemical processing of the rat testis reveals the incidence of apoptosis cells in different treatment groups. TUNEL positive germ cells show apoptotic in D-gal-induced aged rat testis (**B**, arrow). The treatment of vitamin D3 at a dose of 40 IU/Kg to D-gal-induced aged rat also shows a number of TUNEL positive cells, shows apoptosis (**C**, arrow). However other groups showed no TUNEL positive germ cells in rat testis (**A**,**D**–**F**). The figure shows 40X magnifications.

To confirm the role of vitamin D3 on germ cell apoptosis during aging, we also assessed apoptosis of spermatogenic cells by TUNEL (Fig. 4A–F). The results of TUNEL clearly showed apoptosis of germ cell in D-gal-induced aged rat testis, as evidenced by increased TUNEL positive cells in the testis (Fig. 4B). The TUNEL positive cells were observed in the periphery of seminiferous tubules, suggesting apoptosis of spermatocytes. The treatment of vitamin D3 at a dose of 40 IU/Kg to D-gal aged rat showed many TUNEL positive cells in the testis (Fig. 4C). However, very few or no TUNEL positive cells were observed in the testes of the control group (Fig. 4A), treatment of vitamin D3 at a dose of 400 IU/Kg to aged rat (Fig. 4D), and only treatment of vitamin D3 to normal rat (Fig. 4E,F).

### Effect of vitamin D3 on the testicular expression of HSP1A1 and AGER

**Figure 5:**
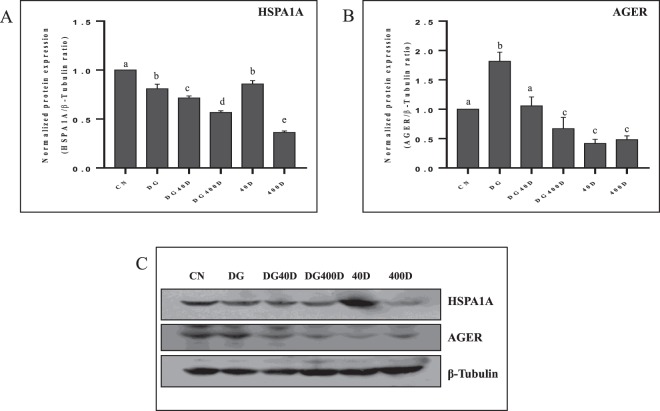
Vitamin D3 modulates the expression of HSP1A1 and AGER in aged rat testis. (**A**) Densitometry of Western blots of HSP1A1 protein expression in rat testis. (**B**) Densitometry of Western blots of AGER protein expression in rat testis. (**C**) Representative Western blots of HSP1A1 and AGER protein expression in rat testis. Values are mean ± SEM (n = 6) and different superscripts show significant (*P* < 0.05) difference in protein expression. CN Control, DG D-galactose, DG40D D-galactose plus 40 IU/Kg vitamin D3, DG400D D-galactose plus 400 IU/Kg vitamin D3, 40D 40 IU/Kg vitamin D3, 400D 400 IU/Kg vitamin D3 groups. Cropped blots are shown. Uncropped blots are presented in Supplementary Fig. S1.

Expression of HSP1A1 showed a significant (*P* < 0.05) decline in D-gal-induced aged rat testis (DG) compared to the control group (CN). Furthermore, treatment of vitamin D3 to D-gal-induced aged rat significantly (*P* < 0.05) decreased expression of HSP1A1 in a dose-dependent manner compared to the control group (CN) and aged rat (DG). The expression of HSP1A1 also showed a significant (*P* < 0.05) decline in normal rat treated with both dose of vitamin D3 (40D and 400D) compared to the control group (CN) (Fig. 5A).

AGER showed significantly (*P* < 0.05) highest expression in testis of D-gal-induced aged rat (DG) compared to other groups. Vitamin D3 treatment significantly (*P* < 0.05) decreased expression of AGER in the testis of D-gal-induced aged rat (DG40D and DG400D) in a dose-dependent manner compared to aged rat testis (DG). Furthermore, vitamin D3 treatment to normal rat at both doses (40D and 400D) significantly (*P* < 0.05) decreased expression of AGER in the testis (Fig. 5B).

### Effect of vitamin D3 on testicular oxidative stress (lipid peroxidation) and antioxidants status (SOD, catalase and GSH)

**Figure 6:**
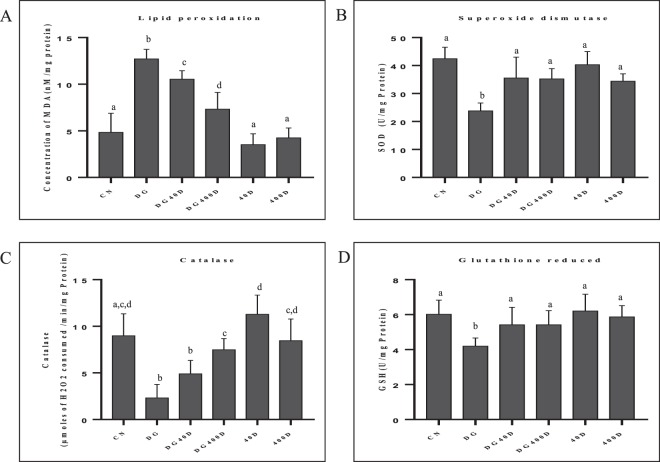
Vitamin D3 mediated regulation of oxidative stress and antioxidant systems (SOD, catalase and GSH) in rat testis. (**A**) Oxidative stress measured as MDA levels and values are expressed nM/mg of protein. (**B**) SOD activity expressed in U/mg of protein. (**C**) Catalase activity expressed as µmole of H_2_O_2_ consumed/min/mg of protein. (**D**) Reduced glutathione (GSH) expressed as U/mg of protein. Values are mean ± SEM (n = 6) and different superscripts show significant (*P* < 0.05) difference. CN Control, DG D-galactose, DG40D D-galactose plus 40 IU/Kg vitamin D3, DG400D D-galactose plus 400 IU/Kg vitamin D3, 40D 40 IU/Kg vitamin D3, 400D 400 IU/Kg vitamin D3 groups.

Changes in MDA levels for lipid peroxidation and activities of SOD and catalase, along with levels of GSH in the testis of rat collected from all groups are summarized in Fig. 6A–D. The levels of MDA was a significantly (*P* < 0.05) increased in D-gal-induced aged rat testis (DG) compared to the other groups. The treatment of vitamin D3 to D-gal-induced aged rat (DG40D and DG400D) significantly (*P* < 0.05) decreased MDA levels in the testis in a dose-dependent manner compared to the aged groups (DG). The levels of MDA in the testis of only vitamin D3 treated groups (40D and 400D) was not statistically different (*P* > 0.05) from the control group (CN) (Fig. 6A).

Antioxidant markers, SOD and GSH also showed a significant decline in D-gal-induced aged rat testis (DG) compared to all other groups. Vitamin D3 treatment at both doses (DG40D and DG400D) to aged rat, significantly (*P* < 0.05) increased the activity of SOD and levels of GSH compared to aged group (DG).

The activity of catalase also showed a significant (*P* < 0.05) decline in the testis of D-gal-induced aged rat (DG) compared to the other groups. Vitamin D3 treatment (DG40D and DG400D) to the aged rat significantly (*P* < 0.05) increased the catalase activity of the testis in a dose-dependent manner compared to the aged group (DG). Vitamin D3 treatment to normal rat (40D and 400D) did not show a significant change in catalase activity compared to the control group (CN) (FIG).

### Effect of vitamin D3 on circulating and intra-testicular levels of vitamin D and AGE

**Figure 7:**
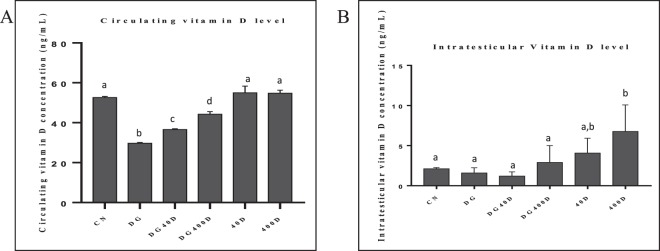
Effect of vitamin D3 on circulating and intra-testicular vitamin D3 levels. (**A**) Circulating vitamin D3 levels showed significant decreased (*P* < 0.05) in D-gal-induced aged rat. (**B**) Intra-testicular vitamin D3 levels showed significant (*P* < 0.05) increased in the testis of normal rat treated with vitamin D3 compared to other groups. Values are mean ± SEM (n = 6) and different superscripts show significant (*P* < 0.05) difference. CN Control, DG D-galactose, DG40D D-galactose plus 40 IU/Kg vitamin D3, DG400D D-galactose plus 400 IU/Kg vitamin D3, 40D 40 IU/Kg vitamin D3, 400D 400 IU/Kg vitamin D3 groups.

The levels of circulating vitamin D3 showed marked variations. The levels of serum vitamin D3 significantly (*P* < 0.05) decreased in D-gal-induced aged rat (DG) and compared with other groups. Furthermore, vitamin D3 treatment to aged rat (DG40D and DG400D) significantly (*P* < 0.05) increased serum vitamin D3 levels in a dose-dependent manner compared to the aged group (DG). However, vitamin D3 treatment to normal rat (40D and 400D) did not show a significant change in circulating vitamin D3 levels compared to the control group (CN) (Fig. 7A). The intra-testicular vitamin D3 did not show marked variation, although the vitamin D3 treatment at 400 IU/Kg (400D) showed significantly (*P* < 0.05) higher levels compared to the other groups (Fig. 7B).

**Figure 8:**
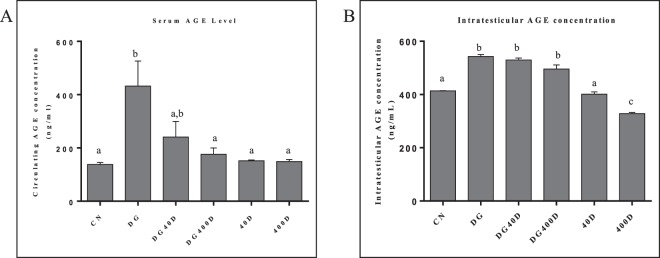
Effect of vitamin D3 on circulating and intra-testicular AGE levels. (**A**) Circulating AGE levels showed significant increased (*P* < 0.05) in D-gal-induced aged rat group and D-gal aged art treated with vitamin D3 40 IU/Kg groups compared to other groups (DG and DG40D). (**B**) Intra-testicular AGE levels showed significant (*P* < 0.05) increased in the testis of DG, DG40D and DG400D compared to other groups. Values are mean ± SEM (n = 6) and different superscripts show significant (*P* < 0.05) difference. CN Control, DG D-galactose, DG40D D-galactose plus 40 IU/Kg vitamin D3, DG400D D-galactose plus 400 IU/Kg vitamin D3, 40D 40 IU/Kg vitamin D3, 400D 400 IU/Kg vitamin D3 groups.

The levels of circulating AGE also showed a significant (*P* < 0.05) high value in D-gal-induced aged rat (DG) compared to the other groups. Vitamin D treatment to the aged rat (DG40D and DG400D) significantly (*P* < 0.05) decreased the serum AGE levels compared to the aged group (DG). The treatment of vitamin D to normal rat (40D and 400D) did not show a significant change in circulating AGE compared to control (Fig. 8A). However, intra-testicular levels of AGE showed significantly (*P* < 0.05) higher value in D-gal-induced aged rat (DG), and the aged rat treated with vitamin D (DG40D and DG400D) compared to control. The treatment of vitamin D to normal rat did not show a significant change in intra-testicular AGE levels compared to control (Fig. 8B).

### Effect of vitamin D3 on the localization pattern of VDR in the testis

**Figure 9:**
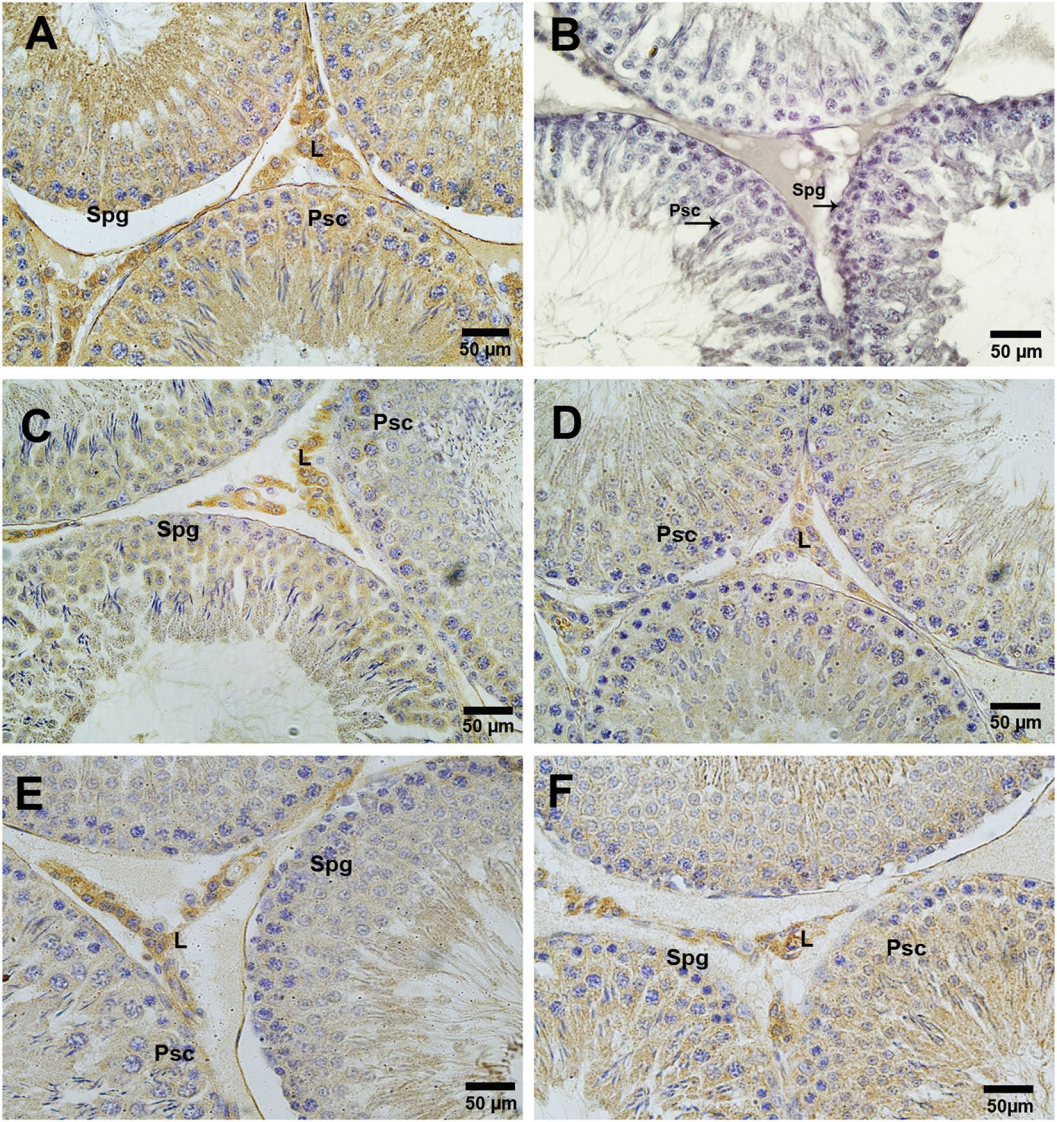
A-F Effect of vitamin D3 on the localization of VDR in the testis of different groups. Control rat showed localization of VDR in Leydig cells (L), Sertoli cells, Spermatogonia (Spg) and Primary spermatocytes (Psc). (**A**) The testis of D-gal-induced aged rat showed no immunostaining of VDR. (**B**) The treatment of vitamin D3 resume the localization of VDR in the testis. (**C**,**D**) The alone treatment of vitamin D3 to normal rat also showed distinct localization of VDR in the testis. (**E**,**F**) The figure showed 40X magnifications.

To study the morphological distribution of vitamin D receptor (VDR) in the testis of all groups, we performed immunohistochemistry of VDR in paraffin-embedded sections. The localization of VDR exhibits marked variation in the pattern. In control rat testis, VDR showed strong immunostaining in the Leydig cells, Sertoli cells, spermatocytes and sperm. Testis of D-gal-induced aged rat showed no immunostaining of VDR. Treatment of vitamin D3 at a dose of 40 IU/Kg to aged rat showed a resumption of mild VDR immunostaining in Leydig cells and spermatocytes as well. Treatment of vitamin D3 to normal rats also showed intense immunostaining of VDR in Leydig cells and spermatocytes (Fig. 9A–F).

## Discussion

The mammalian testis has been evolved to produce male gametes throughout the male life span. The capacity of gamete formation i.e. well functioning of spermatogenesis decides the male fertility. The impairment in spermatogenesis leads to infertility and it has been shown that the spermatogenesis gradually declines with the advancement of age. The dynamics of germ cells in testis is precisely regulated by a balance between continuous cell proliferation and apoptosis^25,26^. It has also been shown that the age-associated imbalance of proliferation and apoptosis results in male infertility^27^. Therefore, prevention or delaying of age-associated germ cell loss would be helpful in managing the fertility in aged male. It has been suggested that during aging the synthesis of vitamin D decline and its deficiency has become a major health concern which causes deterioration of semen quality in male^1,8,28^. However, the role of vitamin D in age-associated decline of testicular functions has been investigated so far. Thus, the present study was aimed to investigate the role of vitamin D3 on aged testis in relation to proliferation and apoptosis.

In the present study we unraveled the effect of vitamin D3 on testicular proliferation markers (PCNA and GCNA), anti-apoptotic and pro-apoptotic marker (BCL2, BAX, active caspase-3), oxidative and antioxidants factors (lipid peroxidation, catalase, SOD, GSH) along with expressions of AGER and HSP1A1 in D-gal-induced aged rat model. The western blot analysis and immunolocalization study showed that expression of cell proliferation markers, PCNA and GCNA decline in the aged testis. The treatment of vitamin D3 clearly showed the expression of PCNA and GCNA increased in aged testis. These results suggest that vitamin D3 stimulates cell proliferation and age-related loss of testicular germ cells could be compensated by vitamin D3 treatment in aged subjects. Vitamin D regulates calcium and bone metabolism, its role in cell proliferation and differentiation have also been documented^29^. Furthermore, it has been shown that vitamin D supplementation to vitamin D3 deficient animal increases the germ cell proliferation by increasing PCNA expression in mice testis^24^. GCNA has also been considered an efficient maker testicular germ cell proliferation and the expression of GCNA decrease with age^30^. Thus, it may be suggested that the age-associated decline of testicular germ proliferation restored by regulating PCNA and GCNA expression by vitamin D3.

As it has been evidenced that apoptosis is programmed cell death, which increases with the advancement of age in the testes of various mammals^31–33^. The results of the present study also showed that the expression of anti-apoptotic factor BCL2 decreased and expression of BAX increased in D-gal-induced aged testis. These findings suggest an increased rate of apoptosis in aged testis. Since we also investigated the expression of active caspase-3 in the testis of D-gal-induced aged rat, this showed that during aging the germ loss is associated with apoptosis. Furthermore, it has been shown caspase activation is required for intrinsic and extrinsic apoptosis in the testis of rat^34^. The previous study also showed that the expression of caspase-3 and BAX significantly up-regulated in natural aging rat testes^35^. The treatment of vitamin D3 to D-gal-induced aged rat showed up-regulation of BCL2 and down-regulation of BAX and active caspase-3 in the testis, which clearly demonstrated that vitamin D3 regulates apoptosis in the testis during aging to prevent germ cell loss and it might be suggested that deficiency of vitamin D3 or improper signaling of vitamin D3 due to down-regulation of VDR in the testis leads to germ cell loss which could, in turn, leads to decrease in fertility. Our immunolocalization study of VDR also showed mild or no staining in D-gal-induced aged rat testis and treatment of vitamin D resume the staining of VDR in testis. Thus, it can be suggested that aged-associated decline of vitamin D receptor could be responsible for increased apoptosis. Since VDR might be localized in the cell membrane or in the nucleus, so further study like electron microscopy would be important to specify the precise localization of VDR during aging testis. To best of our knowledge, the role of vitamin D3 in testicular proliferation and apoptosis has not been investigated. However, the results of the previous study have shown that vitamin D regulates testicular apoptosis in diabetic condition and it has also been demonstrated that vitamin D has pro-apoptotic and anti-apoptotic effects^36–38^. In the present study, TUNEL assay has also shown that during aging testicular germ cell apoptosis increases and treatment of vitamin D3 decrease apoptosis in the aged testis. Earlier reports have also shown that vitamin D is involved in apoptosis and vitamin D deficient animal have decreased sperm count and motility due to decreased proliferation of spermatogenic cells and increased apoptosis of spermatogenic cells^24,36,39^.

Aging is multifactorial processes which decline overall body function and one of the most important factors is free radicals which affect cell constituents and excess of free radicals may lead to cell death^40^. Our result also showed elevated oxidative stress in D-gal-induced aged rat testis. A recent study demonstrated that with age the expression of enzymatic and non-enzymatic antioxidants decreases, which led to increasing damage due to oxidative stress in rats^41^. Moreover, our study reveals that the enzymatic antioxidants, SOD and catalase and non-enzymatic antioxidant GSH decreased in the testis of the aged model. These findings advocate that during aging due imbalance of antioxidant the degenerative changes increases and may lead to decrease spermatogenesis and infertility. It has been documented by the previous study that increased reactive oxygen species and oxidative stress are a major factor for cell apoptosis^42^. The present and previous studies also demonstrated that decreased the activities of SOD, GSH, and catalase, and increased the lipid peroxidation in aged rat testes increase apoptosis. Age-associated increase apoptosis is responsible for the decline of testicular functions^43^. The treatment of vitamin D3 significantly decreased the lipid peroxidation and increased the activities of SOD, catalase and GSH in D-gal-induced aged testis and it can be hypothesized that vitamin D3 mediated improvement in antioxidant status in aged testis might be responsible for decrease apoptosis and increase proliferation of germ cell to protect against age-associated degenerative changes. Whether age-associated testicular dysfunction could be restored or prevented by vitamin D3 in relation to proliferation, apoptosis and antioxidants have not been explored. A recent study, however, shown that vitamin D has potential protective effects in testicular injury through anti-apoptotic and antioxidant pathways^44^. Since the testicular antioxidant and apoptosis and proliferation are well regulated by male hormone testosterone^45,46^ and our recent study showed that levels of testosterone decreased in D-gal-induced aged rat^47^. Thus, it may be hypothesized that vitamin D3 mediated testosterone synthesis could be involved in the regulation of anti-apoptosis and maintenance of antioxidants system in the aging testis. Further research should be conducted to establish the association between aging, oxidative stress and testosterone in the aging testis. The other vitamin, C and E have shown to improve the spermatogenesis by reducing oxidative stress and testosterone production in testis^48^.

Advanced glycation end products (AGEs) include diverse macromolecules, such as carboxymethyl lysine, carboxyethyl lysine, pentosidine, glucosepane, methylglyoxal lysine dimer, glyoxal lysine dimer, and glycolic acid lysine amide, which are synthesized by of non-enzymatic glycation of proteins and lipids^49^. It has been also reported that AGEs accumulation is accelerated during various conditions including aging^50–53^. Our results also showed significantly high levels of AGE in serum and testis in D-gal-induced aged rat. The production of AGE initiates activation of various signaling pathways facilitated by their receptors and the receptor of AGE (AGER) is a well-characterized receptor for AGE signaling^54^. The expression of AGER also increased in D-gal-induced aged rat testis. Furthermore, treatment with vitamin D3 significantly decreased the serum and testicular AGE levels along with the decreased expression of AGER in the aged rat testis. These results suggest that vitamin D3-mediated AGE/RAGE system in aged testis might be involved in the regulation of oxidative stress and control proliferation and apoptosis in aged testis. It has been suggested that vitamin D3 receptor may play important role alterations of the AGE/RAGE pathway chronic kidney disease^55^. The role of vitamin D in testicular AGE/AGER has not been studied during aging, however, it has been shown that vitamin D supplementation mitigates the accumulation of AGEs in the vascular system in rats with streptozotocin-induced diabetes^56^. It has also been demonstrated that AGEs inhibit testosterone synthesis in rat Leydig cells and decrease testosterone production increase oxidative stress by endoplasmic reticulum stress^57^. Thus it appears that AGE/RAGE system regulates oxidative stress and also regulates apoptosis by Bcl-2 and BAX pathway^58^. Since the present study has investigated the role of vitamin D3 in testicular AGE/RAGE system in D-gal-induced aged model, so it would be very interesting to study the role of vitamin D3 in other aged models like natural aging model, senescence-accelerated model, or transgenic model. Furthermore, the *in vitro* study would be very useful to explore the exact role of vitamin D3 in AGE/RAGE system in relation to testicular aging.

The present study also investigated the effect of vitamin D3 on HSP1A1 expression in aged testis and results showed that the expression of HSP1A1 decreased in D-gal-induced aged rat testis as compared to control. It has been shown that HSP1A1 protects cells during hyperthermia and other physiological stresses and expression of HSP1A1 decreases with age at the transcription level^59^. The exogenous treatment of HSP1A1 has also been shown to preserve the age-associated decline of memory and cognitive functions^60^. HSP1A1 has been shown to inhibit apoptosis and also promotes apoptosis by NF-κB^61^. HSP1A1 has diverse biological functions in the regulation of aging and longevity such as oxidation, mitochondrial biogenesis, apoptosis, immunosenescence and inflammation^62,63^. Thus it may be hypothesized that decreased HSP1A1 could promote germ cell apoptosis in aged testis. However, the treatment of vitamin D3 further decreased the HSP1A1 expression in the testis as compared to aged and control. Since vitamin D3 treatment decreased germ cell apoptosis in aged testis along with decreased HSP1A1, which suggests that vitamin D3-mediated down-regulation of HSP1A1 in aged testis might also be involved in decrease apoptosis of germ cell. However, this explanation requires further study to show the more precise role of HSP1A1 in testicular aging.

Our study also showed that serum vitamin D3 significantly decreased in the in D-gal-induced aged rat and vitamin D3 treatment elevated the serum levels of vitamin D3 in aged rats. It has been shown that vitamin D deficiency has caused impaired spermatogenesis with degenerative changes in the rat testis^64,65^. On the other hand, it has also been shown that during aging, circulating vitamin D levels decline and treatment of vitamin D has been shown to improve the levels of male hormones in middle-aged men^66^. A recent study by Dehghani *et al*.^67^ has also shown that vitamin D treatment has a protective role in heart aging in D-gal-induced aged model. Our recent study has also shown that vitamin D3 treatment to D-gal-induced aged rat improved the testicular steroidogenesis^68^.

In conclusion, the present study has shown, for the first time, the role of vitamin D in testicular proliferation and apoptosis during aging. We suggest in D-gal-induced aged testis, the decreased levels of vitamin D3, antioxidants, SOD, catalase, and GSH, while the increased oxidative stress might result in overproduction of ROS, which stimulates apoptosis (increase active caspase-3 and BAX and decreased BCL2) and suppresses germ cell proliferation (decreased GCNA and PCNA). Furthermore, treatment of vitamin D3 significantly suppresses the oxidative stress, which could decrease apoptosis and increase germ cell proliferation in aged testis. Thus, the vitamin D3-mediated regulation of proliferation, apoptosis and antioxidants system in testis during aging might be involving AGER and HSP1A1 in the pathway. These data provide important insights into the application of vitamin D3 in male anti-aging to improve the aged-associated impaired spermatogenesis, although its exact mechanism remained to be further clarified.

## Methods

### Ethics statement

All animals used in this experiment were approved by the Mizoram University Institutional Animal Ethical Committee (MZUIAEC) (approval number: MZUIAEC16-17-10), Mizoram University, Mizoram. All experiments were performed in accordance with relevant guidelines and regulations by the Mizoram University Institutional Animal Ethical committee during the whole experiment.

### Animals and induction of aging

Healthy Adult Wistar albino male rats weighing 150–200 g (3–4 months old) were selected from the inbred colony of Animal Care Facility at the Department of Zoology, Mizoram University, Aizawl, Mizoram, India. All the animals were housed using ventilated sterile polypropylene cages (47 × 34 × 20 cm), and; were provided locally procured sterile sawdust as bedding. All experimental animal groups had access to standard pelleted food and sterile water *ad libitum* and were maintained under controlled temperature (25 °C) and a photoperiod of 12 h light/12 h dark cycle.

For induction of rat aging model, the dose and mode of administration were adopted from the report described by Gao *et al*., 2005^69^. Rats were randomly divided into six (6) groups comprising 6 animals each as follows:

1. Group 1(CN): Animals were given 0.9% saline only.
2. Group 2(DG): Animals were given only D-galactose (120 mg/Kg).
3. Group 3(DG40D): Animals were given D-galactose (120 mg/Kg) plus treatment with vitamin D3 (40 IU/kg)
4. Group 4(DG400D): Animals were given D-galactose (120 mg/Kg) plus treatment with vitamin D3 (400 IU/Kg).
5. Group 5(40D): Animals were given only vitamin D3 (40 IU/Kg).
6. Group 6(400D): Animals were given only vitamin D3 (400/Kg).

D-galactose was given for 42 consecutive days and vitamin D3 treatment was given twice weekly (Tuesday and Friday) for 6 weeks. The doses of vitamin D3 was based on the report described by Han *et al*.^70^. All the animals in the control group received the same amount of normal saline subcutaneously.

### Sample collection

After 24 h of the final treatment, all animals were sacrificed by intraperitoneal injection of Ketamine (90 mg/kg body weight) and Xylazine (10 mg/kg body weight). Whole blood was collected and centrifuged at 1000 × *g* at RT for 10 minutes and serum was collected. Testes were collected by making vertical midline lower abdominal incision. After removing the adherent connective tissues, one testis of each animal was stored immediately at −20 °C and the contralateral testis was fixed in Bouin’s fixative containing 75% of saturated solution of Picric acid, 25% of Formaldehyde and 5% glacial acetic acid at least for 24 h and then transferred to 70% ethanol for later examination. All the dissection procedures were carried out in the aseptic condition.

To prepare total protein lysate, fragments of testes were weighed after removing tunica coverings. The tissue fragments were homogenized in an ice-cold suspension buffer containing 50 mM Tris–Hydrochloric acid, pH 8.0; 150 mM Sodium Chloride (NaCl); 0.1% Sodium Dodecyl Sulfate (SDS); 1 g/ml Aprotinin; 1 mM Phenylmethylsulfonyl fluoride (PMSF) and 1 mM Ethylenediaminetetraacetic acid (EDTA) disodium salt dehydrate (cat# E5134;Sigma–Aldrich,St.Louis,MO,USA) to yield 10% homogenate (w/v). The supernatants were taken after centrifugation at 10000 × *g* at 4 °C for 10 minutes and immediately stored at −20 °C for western blot analysis. For enzyme assay, tissues were homogenized with above-mentioned buffer without SDS.

### Measurement of lipid peroxidation

The Malondialdehyde (MDA) level of the testis was determined according to the principle previously described^71^ with minor modifications^72^. In brief, 75 µl of testis lysates were mixed with the same amount of 10% Trichloroacetic acid (TCA). Freshly prepared 0.2% Thiobarbituric acid was then added in a ratio 1:2 and then boiled for 45 minutes. The solutions were allowed to cool down and the optical densities were measured against the blank at 532 nm and the concentration of MDA was expressed as nM/mg protein. A mixture devoid of testis protein lysate served as blank.

### Superoxide dismutase (SOD) assay

The previously described method was adopted for the superoxide dismutase assay^73^. Briefly, a mixture containing 50 µl of 10% tissue homogenate, 600 µl of 52 mM sodium pyrophosphate buffer (pH 8.3), 50 µl of 186 µM Phenazine methosulphate (PMS), 150 µl of 300 µM Nitroblue tetrazolium(NBT). The reaction was started by the addition of 100 µl of 750 µM ml NADH and incubated at 30 °C. The reaction was terminated after 90 seconds incubation by the addition of 500 µl glacial acetic acid. Two ml of n-butanol was added, vortexed and allowed to stand for 10 minutes. The mixture was centrifuged at 10000 × *g* for 10 minutes at room temperature and the supernatant was collected. Color intensity was measured at 560 nm and the concentration of SOD was expressed as units (U)/mg protein. A mixture devoid of tissue homogenate was taken as blank.

### Glutathione reduced (GSH) assay

An earlier described principle and method was adopted for Glutathione reduced assay^74^. Briefly, A mixture containing 2.0 M Pyrophosphate buffer(pH.7.4), 0.2 M of 5,5′-dithio-bis-[2-nitrobenzoic acid], and 50 µl of 10% tissue homogenate. The assay was based on the rate of formation of TNB-chromophore from the reaction of DTNB with intracellular GSH content. The TNB complex has a maximum absorbance of 412 nm. The TNB complex formation rate was considered proportional to the GSH content of the tissue samples and the concentration of GSH was expressed as U/mg protein. The reaction mixture devoid of tissue homogenate served as blank.

### Catalase assay

A previously described method was adopted for the determination of Catalase activity^75^. Briefly, the assay mixture containing 1 ml of PBS (pH 7), 100 µl of testis tissue lysate and 400 µl of 2 M hydrogen peroxide (H_2_O_2)_ was incubated for 1 minute and the reaction was stopped by the addition of 2.0 ml dichromate-acetic acid reagent (5% potassium dichromate and glacial acetic acid in a ratio of 1:3). Using spectrophotometer (Eppendorf BioPhotometer) the absorbance was measured at 570 nm. The activity was expressed as µM of H_2_O_2_ consumed per minute per mg of protein (H_2_O_2_ consumed/minute/mg protein). A mixture devoid of tissue homogenate was taken as blank.

### Western blotting

Testicular protein lysates were subjected to western blot analysis as described earlier^47^. In brief, the total protein concentration of each lysate was evaluated by employing Bradford methods^76^. The equal amounts of proteins (50 µg) were electrophoresed using 10% sodium dodecyl sulfate – Polyacrylamide gel electrophoresis (SDS-PAGE) at 150 V. The resolved proteins were transferred onto polyvinylidene difluoride (PVDF) membrane by Medox-Bio Mini SemiDry Blotting MX-1295-01 apparatus for 20–30 minutes. Membranes were then blocked in blocking solution containing 0.05% Tween 20 in Phosphate-buffered saline (PBS) and 10% non-fat skimmed milk (Cat# GRM1254-500G HiMedia Laboratory private limited, Mumbai, India) for at least 1-h at room temperature. The membranes were then probed with BCL2 (1:1000, Rabbit polyclonal antibody, Cat# EPP10828 (New Cat#E-AB-22004), Elabscience, Wuhan, China), BAX (1:1000, Rabbit polyclonal IgG, Cat# SC-6236, Santa Cruz Biotechnology Inc. Dallas, USA), Anti-Active Caspase-3 (1:1000, Mouse monoclonal antibody, Cat# STJ97448, St John’s Laboratory, London, UK) Elabscience, Houston, Texas, USA), Proliferating cell nuclear antigen (PCNA) (1:1000, Rabbit polyclonal IgG, Cat# SC-7907, Santa Cruz Biotechnology Inc. Dallas, USA), Germ cell nuclear antigen (GCNA) (1:1000, Mouse monoclonal IgG, Cat# 10D9G11, Developmental Studies Hybridoma Bank University of Iowa, Department of Biology, Iowa), Advanced Glycation End Product receptor (AGER) (1:1000, Cat#EPP10244 (New Cat#E-AB-32739), Elabscience, Wuhan, China), Heat shock protein primary antibody (HSP1A1) (1:1000, mouse monoclonal IgG, Cat# SC-32239, Santa Cruz Biotechnology Inc. Dallas, USA) for overnight inside humidified chamber at 4 °C. After PBST wash, membranes probed with rabbit raised primary antibody were incubated with Horse-radish Peroxidase (HRP) conjugated goat-anti-rabbit secondary IgG antibody (1:4000, Merck Specialties Pvt. Ltd, Mumbai, India) and membranes probed with mouse raised primary antibody were incubated with HRP conjugated goat-anti-mouse secondary IgG antibody (1:4000, Cat# SC-2005, Santa Cruz Biotechnology Inc. Dallas, USA) for 3-h with constant agitation at room temperature. The membranes were washed twice again with PBST and then immersed in Enhanced Chemiluminescence (ECL) solutions (Cat #1705061, BioRad, Hercules, CA, USA) as per the manufacturer’s instructions and then developed onto X-ray film in a dark room. The band intensities were quantified by using ImageJ software. A separate probe was developed for β-Tubulin which served as a loading control and all the antibodies used in this experiment were normalized against it.

### Immunohistochemistry

Testis tissues were dehydrated by directly immersing in the subsequent increasing concentration of ethanol (70%, 90% and 100%) for 1-h each so as not to excessively distort the tissues. Fully dehydrated tissues were embedded in paraffin by using molten paraffin infiltration method. Tissue block was made and sectioned at 5 µm thick using Leica rotary microtome (model RM2125 RTS). Using floatation bath (Equitron medica private limited, Mumbai, India), the tissue sections were attached to positively-charged hydrophilic glass slides (PathnSitu Cat#PS016-72) and stored at room temperature for later use. Using xylene, the stored tissue embedded slides were deparaffinized and were rehydrated by subsequent decreasing concentrations of ethanol (100%, 90% and 70%) for 10 minutes each. Slides were immersed in PBS for 10 minutes and blocked with Goat serum 1:100 (Santa Cruz Biotechnology, Inc. CA, USA) with PBS inside the humidified chamber for 30 minutes in room temperature. Using primary antibodies; PCNA (1:100, rabbit polyclonal IgG, Cat # SC-7907, Santa Cruz Biotechnology Inc. Dallas, USA), GCNA (1:100, mouse monoclonal IgG, Cat# 10D9G11, Developmental Studies Hybridoma Bank University of Iowa, Department of Biology, Iowa) vitamin D receptor (VDR) (1:100, rabbit polyclonal IgG, Cat # bs-2987R, Bioss Antibodies Inc. Woburn, MA, USA), the slides were incubated overnight at 4 °C in humidified chamber. After rinsing the slides with PBS, the slides were incubated with secondary antibodies; HRP-conjugated goat-anti-rabbit (1:500, Merck Specialties Pvt. Ltd, Mumbai, India) (for PCNA and VDR probed slides), and HRP-conjugated goat-anti-mouse IgG (1:500, Cat# SC-2005, Santa Cruz Biotechnology Inc. Dallas, USA) (for GCNA probed slides) for 3-h at room temperature. The slides were then washed 3 times (5 minutes each) and then incubated with 0.05% Diaminobenzidine (DAB) solution (3, 3’- diaminobenzidine in 5 nM Tris pH 7.6) for 5 minutes. After hematoxylin counterstaining for 5 minutes, the slides were then immersed in tap water, tissues were dehydrated using the increasing concentration of ethanol (70%, 90% and 100%) for 10 minutes each, cleared in xylene and mounted with DPX mountant. The slides were examined and photographed using a Nikon binocular microscope (Model E200, Nikon, Tokyo, Japan).

### Terminal deoxynucleotidyl transferase dUTP nick end labeling (TUNEL) assay

Using Apo-Bru-IHC *In situ* DNA fragmentation assay kit (Catalog #403-50. BioVision Inc. Milpitas, CA, USA), the fixed-stored testis tissues were processed for TUNEL assay according to the manufacturer’s instructions. Briefly, after deparaffinization and rehydration of the tissue, the slides were immersed in PBS and incubated with proteinase K (1:100 in 10 mM Tris pH 8) for 20 minutes at room temperature. The slides were again rinsed with PBS. Endogenous peroxidase was quenched off by incubating the slides in methanol + H_2_O_2_ solution (30% H_2_O_2_ 1:10 in methanol) for 5 minutes. The sections were incubated with labeling reaction mixture (5X reaction buffer 50 µl, terminal deoxyribonucleotidyl transferase (TdT) enzyme buffer 3.75 µl, Bromodeoxyuridine triphosphate (Br-dUTP) solution 40 µl and 161.25 µl double-distilled water) at 37 °C for 1-h. After rinsed with PBS, the slides were blocked with blocking buffer for 10 minutes and immediately incubated with antibody solution (Anti-BrdU-Biotin antibody diluted with blocking buffer in a ratio 1:19) for 1–1.5 h in dark humidified chamber. The slides were then rinsed again with PBS and incubated conjugate solution (200X conjugate mixed with blocking buffer in a ratio 1:200) at room temperature for 30 minutes. The slides were washed in BPS and then incubated with 3, 3′-diaminobenzidine (DAB) solution for 15 minutes. Slides were rinsed with water and counterstained with methyl green solution for 3 min. The slides were subsequently dehydrated in a series of increasing concentration of ethanol (70%, 90% and 100%) for 10 minutes each, cleared in xylene and mounted with mounting media. The TUNEL-positive apoptotic cells were observed as brown cells.

### Measurement of circulating and intratesticular advanced glycation end product (AGE) by ELISA

Enzyme-linked immunosorbent assays were performed as directed by the manufacturer’s instructions. In brief, fragments of testes samples were homogenized using PBS (pH 7.6) in a ratio 1:9 (w/v). 50 µl of both serum and tissue samples were loaded and 50 µl of biotinylated AGE antibody solution was added to each well. After 45 minutes incubation at 37 °C, the contents were decanted and washed 3 times with 1X wash buffer. A working solution of 100 µl HRP-conjugate solution was loaded to each well and incubated in 37 °C for another 30 minutes. Washing was done 3 times with wash buffer and 90 µl of substrate reagent was added and incubated for another 15 minutes at 37 °C. 50 µl stop solution was added and optical density of each well was determined by taking the absorbance at 450 nm using microplate ELISA reader (Erba Lisa Scan EM, TransAsia Biomedical Ltd, Mumbai, Maharashtra, India).

### Measurement of circulating and intratesticular vitamin D3 by ELISA

Intratesticular and serum total vitamin D3 concentration were assayed by using quantitative 25OH Vitamin D ELISA kit (Cat # DKO146) as per the manufacturer’s instructions. Briefly, 10 µl of both serum and tissue homogenate from each group were loaded into each well and incubated for 90 minutes after the addition of 200 µl of working Vitamin D-Biotin Conjugate solution at room temperature. The contents were aspirated off and washed 3 times with 1X wash buffer. 200 µl of Streptavidin-HRP Conjugate was loaded and incubated for 30 minutes at room temperature. The wells were washed again 3 times with 1X wash buffer. 200 µl of TMB Substrate was loaded and incubated in dark for 30 minutes at room temperature. The reaction was stopped by addition of 50 µl stop solution and the absorbance was taken at 450 nm using microplate ELISA reader (Erba Lisa Scan EM, TransAsia Biomedical Ltd, Mumbai, Maharashtra, India). The precision levels for both inter and intra-assay variability was ≥6.95% and ≥6.4% respectively.

### Statistical analysis

Using GraphPad Prism, all statistical analyses were performed. The normal distributions of all the parameters were analyzed by the Shapiro-Wilk normality test. One-way Analysis of variance (ANOVA) followed by Tukey test was used to compare the data from different groups. Data were expressed in terms of mean ± SEM and were considered to be significant if ‘*P*’ value is less than 0.05 (*P* < 0.05).

## Supplementary information

Supplimentary info
