# Supplementary material for: Vitamin D3 regulates apoptosis and proliferation in the testis of D-galactose-induced aged rat model
Source: Sci Rep. 2019 Oct 1;9:14103. doi: 10.1038/s41598-019-50679-y (PMC6773724; doi:10.1038/s41598-019-50679-y)
Supplement: Supplementary file 1 — Supplimentary info [file 41598_2019_50679_MOESM1_ESM.pdf]

Supplementary Information

Vitamin D3 regulates apoptosis and proliferation in the testis of D-galactose induced aged rat model

Malsawmhriatzuala Jeremy, Guruswami Gurusubramanian and Vikas Kumar Roy*
Department of Zoology, Mizoram University, Aizawl, Mizoram - 796 004, India

*Correspondence

Vikas Kumar Roy,
Department of Zoology,
Mizoram University,
Aizawl, Mizoram - 796 004,
India.
Tel: +918794821930
Fax: +913892330644
Email: vikasroy4araria@yahoo.co.in

Supplementary Figure S1 - full-length blots of figure 1

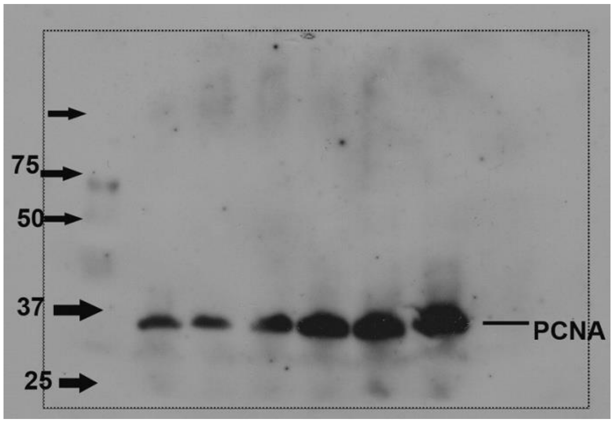

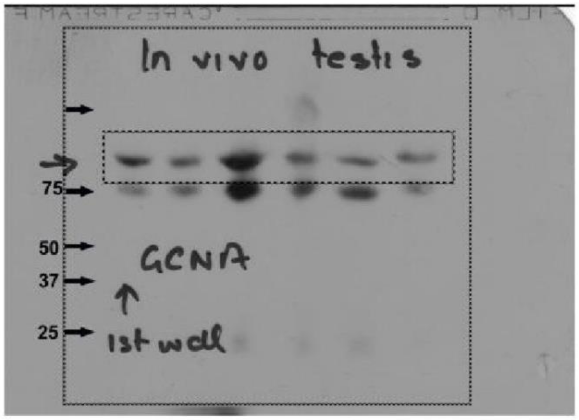

PCNA

GCNA

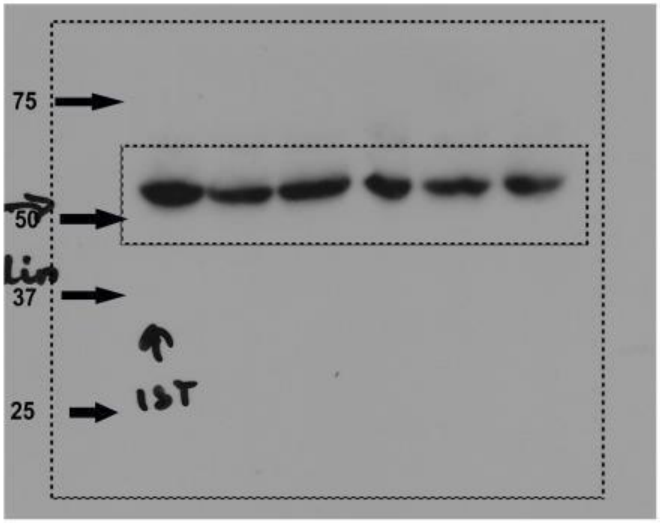

β-Tubulin

Supplementary Figure S1 - full-length blots of figure 3

Bcl2

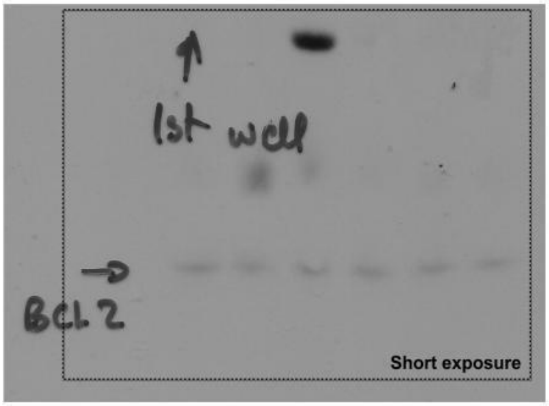

Bcl2

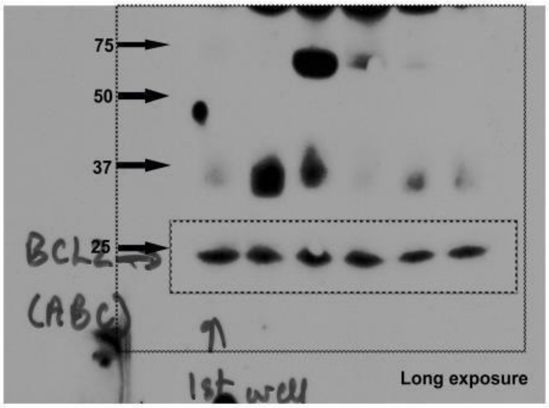

Bax

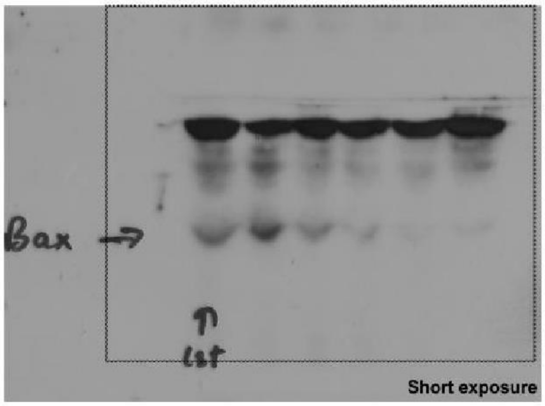

Bax

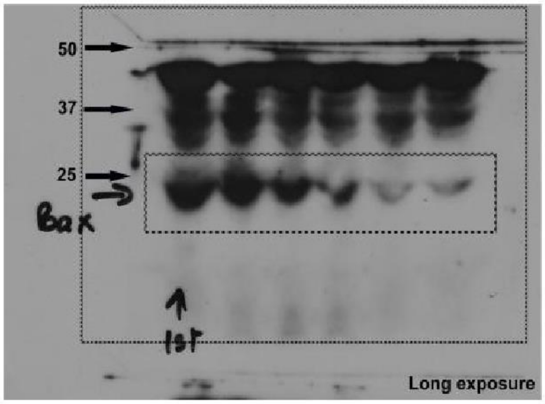

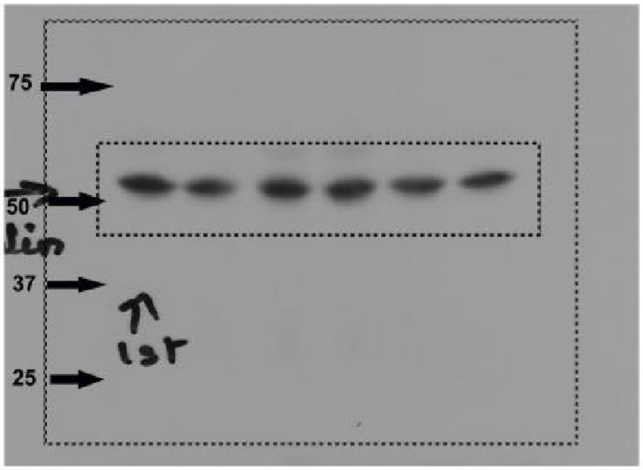

β-Tubulin

Supplementary Figure S1 - full-length blots of figure 5

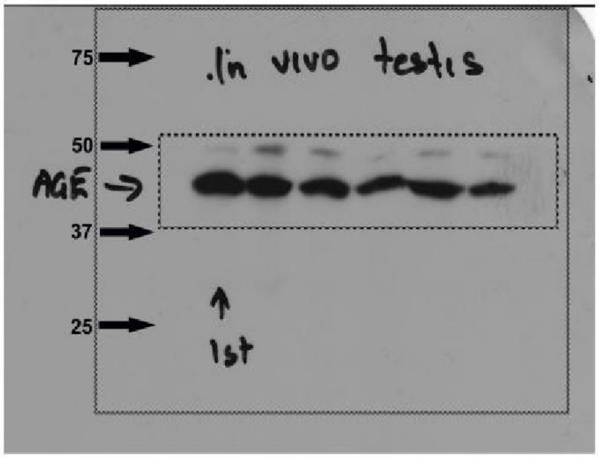

AGER

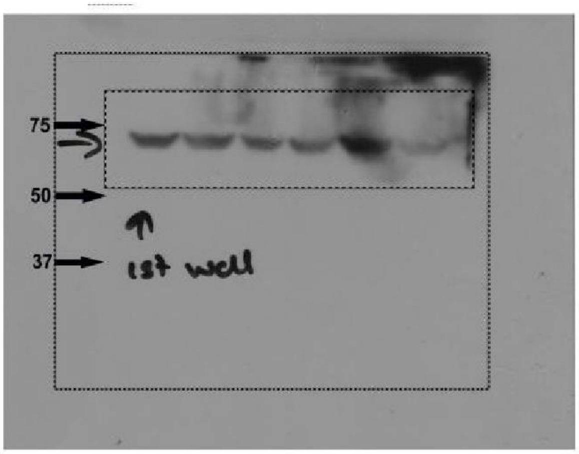

HSP70

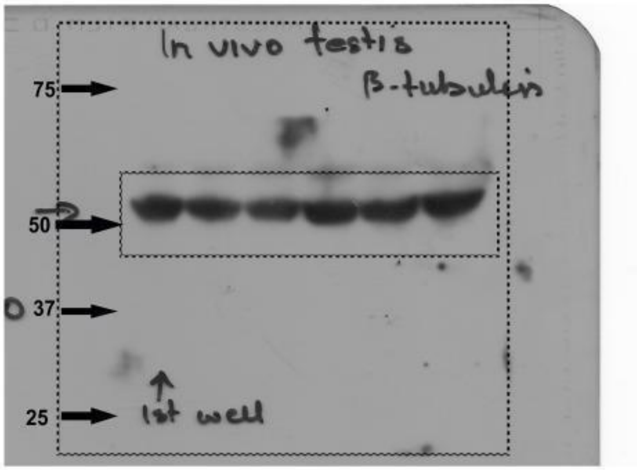

β-Tubulin

Supplementary figure S1 : Validation of Primary antibodies

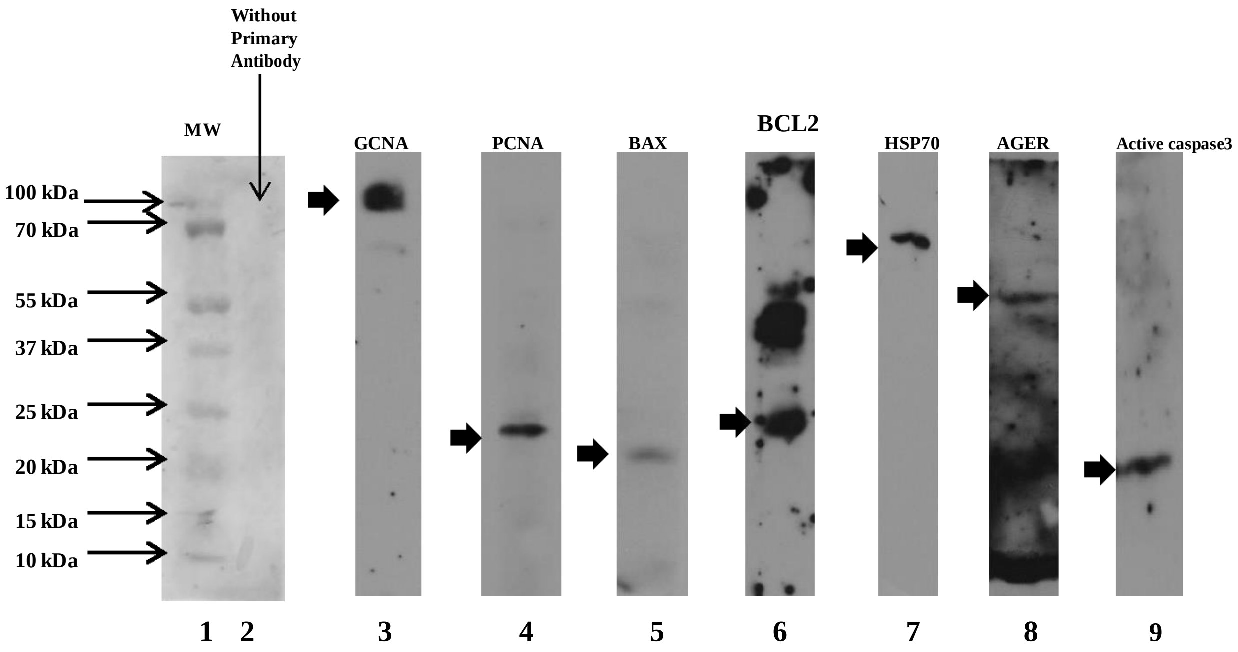

Lane 1- Protein marker. Lane 2- Probed without primary antibody (only Secondary antibody). Lane 3-9 : probed with GCNA,PCNA, BAX,BCL2,HSP70,AGER and Active caspase3 antibodies. All bands are indicated by arrows
